# A Review of Density Functional Models for the Description of Fe(II) Spin-Crossover Complexes

**DOI:** 10.3390/molecules25215176

**Published:** 2020-11-06

**Authors:** Anton Römer, Lukas Hasecke, Peter Blöchl, Ricardo A. Mata

**Affiliations:** 1 Institut für Physikalische Chemie, Universität Göttingen, Tammannstrasse 6, 37077 Göttingen, Germany; aroemer@gwdg.de (A.R.); lukas.hasecke@stud.uni-goettingen.de (L.H.); 2 Institut für Theoretische Physik, Technische Universität Clausthal, Leibnizstraße 10, 38678 Clausthal-Zellerfeld, Germany; peter.bloechl@tu-clausthal.de; 3 Institut für Theoretische Physik, Universität Göttingen, Friedrich-Hund-Platz 1, 37077 Göttingen, Germany

**Keywords:** spin crossover, transition metals, DFT

## Abstract

Spin-crossover (SCO) materials have for more than 30 years stood out for their vast application potential in memory, sensing and display devices. To reach magnetic multistability conditions, the high-spin (HS) and low-spin (LS) states have to be carefully balanced by ligand field stabilization and spin-pairing energies. Both effects could be effectively modelled by electronic structure theory, if the description would be accurate enough to describe these concurrent influences to within a few kJ/mol. Such a milestone would allow for the *in silico*-driven development of SCO complexes. However, so far, the *ab initio* simulation of such systems has been dominated by general gradient approximation density functional calculations. The latter can only provide the right answer for the wrong reasons, given that the LS states are grossly over-stabilized. In this contribution, we explore different venues for the parameterization of hybrid functionals. A fitting set is provided on the basis of explicitly correlated coupled cluster calculations, with single- and multi-dimensional fitting approaches being tested to selected classes of hybrid functionals (hybrid, range-separated, and local hybrid). Promising agreement to benchmark data is found for a rescaled PBE0 hybrid functional and a local version thereof, with a discussion of different atomic exchange factors.

## 1. Introduction

Spin-crossover (SCO) complexes are molecules which exhibit spin transitions under external stimuli such as temperature and pressure, commonly based on d^4^ to d^7^ transition metals. Some of the most notable examples for SCO materials are based on Fe(II) [1], whereby the transition occurs between the S = 0 and the S = 2 states. These complexes have a wide potential application as molecular switches for use in display, memory and sensing devices [2,3,4,5]. Single center complexes make up for a large portion of these materials, since they can exhibit abrupt, well-defined transitions. Supramolecular complexes combining several metal centers have been also developed over the last few years [6,7,8]. The latter have gathered some interest given their unique SCO properties, allowing for hysteretic and multistep transitions.

There have been a multitude of theoretical studies on the spin energetics of Fe(II) complexes. With the inherent difficulties in obtaining a balanced description of the different spin states, internal references are often adopted [9,10]. Hereby, calculations are carried out not only on the target of study but also a reference compound. The theoretical values are then discussed looking at trends instead of absolute values, how the relative energy placement of the two states is shifted by coordination or structural changes. This might be a viable approach for closely related families of compounds, but its robustness is hard to verify *a posteriori*.

It is not clear which theoretical approaches can be reliably applied to simulate the energetics of Fe(II) SCO complexes. The discussion is definitely impaired by the problems in defining suitable benchmark data. Experimental measurements will always include some sort of environment effect [11] and a fully converged electronic structure description is unattainable. Several studies in the past have applied multi-reference approaches including RASSCF/RASPT2, CASSCF/CASPT2, NEVPT2 and SORCI [12,13,14,15,16,17]. Other studies have relied instead on the coupled cluster series, building upon a single-reference picture. All the aforementioned studies have aimed at a convergence of the N- and one-particle spaces for small models of functioning SCO complexes. Here we try to highlight some of the conclusions made over the last few years. Divergence between CASPT2 values [13] and singlet-quintet gaps computed at the CCSD(T) level [18] have been noted in the past and attributed to a failure in the single-reference description of coupled cluster. This is something which needs to be assessed system by system, as the weight of different configurations (mostly determined by the different occupations on the metal d-orbitals) will depend on the specific ligand. However, it should be noted that even in case multi-reference becomes an issue, it is not clear which of the methods will provide the most accurate prediction. The impact of triple excitations is sizeable when comparing different spin states. This is directly observed in the comparison between CCSD and CCSD(T) values of several works [19,20]. Second-order perturbation theory (even in a multi-reference framework) might not capture all the dynamical correlation differences upon spin change. In the end, it is a question of balance between the two flavors of correlation [21]. This is also an issue when it comes to the selection of the electron space to be expanded in correlated calculations. Pierloot and coworkers [14] highlighted in 2017 the importance of electronic correlation effects from the (3s3p) electrons. Also clear in the study is the strong basis set dependence in spin gap energetics.

Albeit scarcer, there are also calculations based on diffusion Monte Carlo (DMC) [22,23,24]. One should note that these show much larger discrepancies among them than the error bounds estimated for their convergence. Overall, in comparison to coupled cluster, DMC appears to stabilize the high-spin states, although the magnitude of the effect changes with the details of the calculation.

Recently, Phan et al. [25] have suggested a strong correlation between the spin state of homoleptic diimine complexes of Fe(II) and the N-N distances in diimine ligands. A target region was identified around 2.8–2.9 Å, whereby magnetic bistability would be likely. The model focuses on the role of orbital overlap in the coordination to the metal, but ignores the electron donating capacity of the chelating ligand, assuming that the main identifier is the imine character of the coordination center. Environment effects are also neglected [26,27]. Albeit simple, the model has a quite surprising predictive power for this class of compounds. This also establishes a clear priority for the development of theoretical screening approaches for SCO complexes. A reliable computational protocol should not only be able to describe quantitatively the HS-LS gap for a specific coordination environment, but also how the latter changes with the relative placement of the coordinating atoms. Studies carried out so far on the benchmarking of DFT [24,28,29] have looked at different ligands, but not upon structure changes which affect the coordination to the metal. The uncertainty about nondynamical effects, and therefore the lack of reference values, has also hampered such efforts.

In this work, we set out to establish whether hybrid density functionals can be adequately parameterized to capture the effects of ligand field stabilization under different geometric constraints. This perspective is orthogonal to most benchmark studies so far, where reference values are computed and compared changing the chemical nature of the compound. We make use of the simplest model system for an Fe(II) complex with N-coordinating ligands, the [Fe^II^(NH_3_)_6_]^2+^ species. We establish a set of standard benchmark tests which can be easily expanded to other centers and varying coordination, focusing on the ligand structure. The fundamental quantity of interest is the adiabatic HS-LS energy difference $\Delta E_{\mathrm{HL}}=E_{\mathrm{HS}}\left(R_{\mathrm{HS}}\right)-E_{\mathrm{LS}}\left(R_{\mathrm{LS}}\right)$, with $E_{\mathrm{XS}}\left(R_{\mathrm{XS}}\right)$ representing the energy of the HS/LS states in their respective geometries. Only electronic energies will be discussed. Environments effects (solution, solid-state interactions) are not discussed but could be introduced on top through standard embedding procedures.

## 2. Results

As mentioned earlier, there is an active discussion about the single reference character of both singlet and quintet states of the [Fe^II^(NH_3_)_6_]^2+^ complex. Such arguments are in line with the many observed difficulties in converging the level of theory for non-heme iron complexes (for a recent example and some selected references see Ref. [30]). Song et al. [24] suggested that the system would have a significant amount of static correlation based on their analysis of the single excitation amplitudes. Two diagnostics are commonly used, the T1 diagnostic which is based on the Frobenius norm and D1, based on the matrix 2-norm [31]. Their computed values varied between 0.049 and 0.087, depending on the spin state and basis set combination. This would be above what was suggested to be a safe threshold of 0.04 [31]. However, such thresholds have been derived from small molecule calculations with no transition metals featured. More recent assessments [32], specifically for 3-d transition metal compounds, place the threshold at D1 < 0.15. Care should also be taken since it is best to combine several diagnostics for a more robust assessment. Wilson and coworkers suggested the combined use of D1 with T1 (should remain below 0.05) and a computed percentage of the atomization energy.

We have carried out calculations on the DFT optimized [Fe^II^(NH_3_)_6_]^2+^ complex to verify these multi-reference descriptors. Our CCSD D1 diagnostics (triple-zeta quality basis set) are even slightly higher (0.092 for the LS and 0.045 for the HS state) than those of Song et al., but still well below 0.15. The T1 values are again below the threshold (0.02 for the LS and 0.013 for the HS state, compared to the suggested value of T1 < 0.05). Having fulfilled two out of three criteria, we would agree with the assessment made by Flöser et al. [33] and deem the multi-reference character of both states to be amenable.

It should also be noted that in a recent report, Radoń benchmarked different electronic structure methods against experimentally derived iron spin-state energetics, finding that CCSD(T) in fact performs remarkably well [28]. Compared to the proposed back-corrected experimental data set, the mean absolute error was about 1 kcal/mol (depending on the choice of reference orbitals). Other comparisons to experimental data have also been favorable to CCSD(T) [14,19].

The convergence at the coupled cluster level of $\Delta E_{\mathrm{HL}}$ for the relaxed geometry of the hexaamino complex has been already extensively addressed by Flöser et al. [33]. Here, we will just provide a small review and compare their results, obtained with local correlation approaches and basis set extrapolation to our own, which are derived from canonical CCSD(T) with explicit correlation.

First of all, we confirmed the additivity of the scalar relativistic corrections to the HS-LS gap at the coupled cluster level. The results are presented in Appendix A. For the hexaamino complex (in its minimum geometry) the latter account for 2.51 kcal/mol, and have a negligible effect on the computed correlation energies. The scalar relativistic corrections can be computed at the Hartree–Fock level and added to non-relativistic CCSD(T) values with little to no effect on the value of $\Delta E_{\mathrm{HL}}$. We applied canonical coupled cluster singles and doubles with perturbative triples (CCSD(T)), with explicit correlation (F12B) and scaled triples. The basis set used was of triple-zeta quality, which should provide close to converged results. Further details are provided in Materials and Methods. Comparison to the aforementioned results of the Neese group show only very small deviations. Our computed scalar relativistic corrections amount to 2.51 kcal/mol, compared to 2.37 kcal/mol. Also in the basis set correction, moving from triple-zeta to CBS extrapolation or in our case using the F12B value as the limit, the deviations are quite small. The CBS[Q:5] correction of Flöser et al. amounts to −4.7 kcal/mol, while our value is at −5.0 kcal/mol. We obtained for [Fe^II^(NH_3_)_6_]^2+^ a value of $\Delta E_{\mathrm{HL}}$ = −13.37 kcal/mol, compared to −11.3 kcal/mol. The difference between their $\Delta E_{\mathrm{HL}}$ result and our value is mostly due to the difference in the geometry optimization.

### 2.1. Benchmark Results

**Figure 1:**
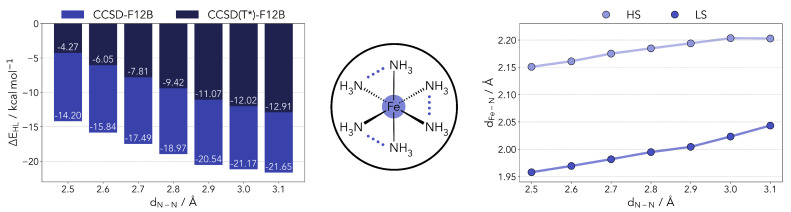
Left panel: Computed HS-LS gap for the [Fe^II^(NH_3_)_6_]^2+^ complex with varying N-N distances at the coupled cluster level (including the DK correction and the scaled triples). Center panel: Diagram of the model complexes used in the benchmark. The N-N distances were restricted pairwise with all other geometry parameters being optimized for both HS and LS states. Right panel: variation in the Fe-N distance as a function of the restraint placed on the ligands.

Taking the work of Phan et al. [25] as a starting point, we built models mimicking different ligand geometries. This was achieved by setting constraints to the N-N distances in the [Fe^II^(NH_3_)_6_]^2+^ model. Given that many SCO complexes of interest bear bidentate ligands, and in keeping with the model of Phan et al. [25], we restrained the distances between the NH_3_ molecules pairwise (see Figure 1). No symmetry constraints were used. The N-N distance range was set between 2.5 and 3.1 Å, effectively covering the predicted optimal SCO range and beyond. The complex geometries were optimized for all other degrees of freedom at the PBE0r [34] level of theory (as described in Materials and Methods). This local hybrid functional is also discussed later in the text.

**Figure A1:**
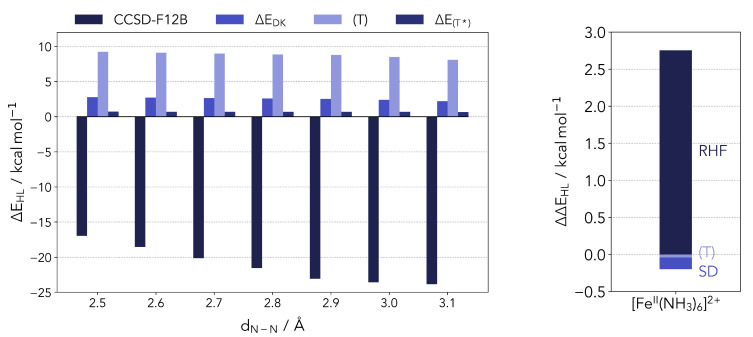
(**Left panel**) different energy contributions to the UCCSD(T*)-F12B reference. (**Right panel**) energy difference between a relativistic and non-relativistic CCSD(T) calculation for the reference complex.

The $\Delta E_{\mathrm{HL}}$ were computed at the coupled cluster level following the procedure described in the previous section. We observed no significant changes in both the T1 and D1 diagnostics over the range studied. The triples correction significantly favors the LS state, decreasing the gap by about 10 kcal/mol. As one can observe, the restraints placed in the ligands contribute to a significant lowering of the $\Delta E_{\mathrm{HL}}$, from −12.9 kcal/mol down to −4.3 kcal/mol. In the range applied, however, it is not sufficient to overturn the stability of the high-spin state. It should be noted that the N-N distances of the fully relaxed model system are around 3.1 and 2.8 Å, for the HS and LS states, respectively. In the Appendix A, Figure A1, we present a breakdown of different energy components for the calculation of $\Delta E_{\mathrm{HL}}$ as a function of the N-N distance. Some terms show a geometry dependence, most notably the CCSD correlation energy and the triples correction. The scaling of the triples (T*) seems to bear little impact.

Taking a closer look at the optimized geometries (right side panel of Figure 1), the shortening of the N-N distance also leads to a shorter coordination distance. The effect is slightly more pronounced in the LS geometries. This relation is determined by the overlap of the nitrogen lone pairs and the Fe e_g_ orbitals. Shortening the distance between the coordinating nitrogens, the orbital overlap can be partly maintained by shortening the Fe-N distance as well, and vice versa. The S = 0 state is less destabilized by this change as it will be able to more easily accept density in the e_g_ levels.

### 2.2. Parameterization of Hybrid Functionals

The main focus of this work is to verify whether it is possible to parameterize hybrid density functionals to correctly replicate the energy gap between the two spin states of interest in Fe(II). We attest this for our model system, with varying N-N distances in the coordination, to be sure that the approach is robust relative to structural changes in the complex. In a sense, we are simulating a large range of ligands with a small number of reference calculations. Optimally, the results should not be degraded upon subtle variations in the coordination geometry. This is a necessary condition to accurately model the dynamics of SCO complexes and to guarantee the predictive power.

We start by setting a baseline, reporting on the results of two GGA (PBE [35], BP86 [36,37]) and two meta-GGA functionals (TPSS [38] and M06-L [39]). All DFT calculations in this work have been performed with Grimme’s proposed D3 dispersion correction [40,41], with the exception of M06-L. In order to avoid too much cluttering in the names, we have dropped the D3 suffix throughout. As it has been repeatedly reported in the literature (for example in Ref. [42]), the stability of the LS states is grossly overestimated by GGA functionals. Nonetheless, many of the calculations on SCO complexes are still carried out at this level of theory. One primary reason is, of course, the lower computational cost. The other advantage is that it also often guarantees that the ground-state will be a LS state. Entropy favors the HS state (longer bonds) so actual SCO complexes where the transition occurs by heating up the system will require a small energy difference between the two, but with the LS state being lower. The difficulties are especially aggravating for multicentered complexes, where benchmarks are even scarcer. For example, in the case of the Fe-grid complexes synthesized at the Meyer group [8], a number of theoretical studies have accurately predicted, in line with magnetic susceptibility measurements that the LS states are indeed more stable [43,44]. However, the energy differences reported between the different spin states are generally too large for an SCO-capable complex. Theory, in this case, provides only a qualitative picture of the process.

**Figure 2:**
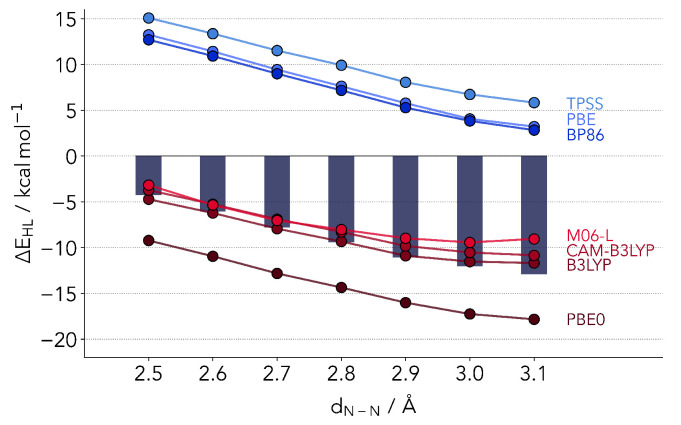
Overview of the DFT $\Delta E_{\mathrm{HL}}$ in relation to the coupled cluster values (bars). In absolute values the hybrid functionals as well as M06-L outperform the remaining GGA and meta-GGA functionals. However, TPSS, BP86 and PBE0 correctly reproduce the coupled cluster trend of a further lowering of the singlet-quintet gap at larger N-N distances.

The results for all four functionals are provided in Figure 2, together with the coupled cluster reference and computations from the other classes of functionals later discussed in the text. The simpler GGA functionals agree rather well among each other, but deviate strongly from the reference. As expected, the LS is much too stable. The same pattern is observed for TPSS. The only outlier is M06-L, which performs in absolute terms much better than its counterparts. However, already one effect is made clear by our selection of benchmark setup. The coupled cluster values predict an almost linear relationship between $\Delta E_{\mathrm{HL}}$ and $d_{\text{N-N}}$. This is well-reproduced by all GGA and meta-GGA functionals, with the exception of M06-L, which flattens out by $d_{\text{N-N}}\geq 3.0$ Å. Nonetheless, the results partly support the comparison of SCO complexes based on GGA, given that the slope is almost the same as the reference values. This in turn signals the possibility of reproducing trends across different ligands or distortions.

#### 2.2.1. Common Hybrid Functionals

**Figure 3:**
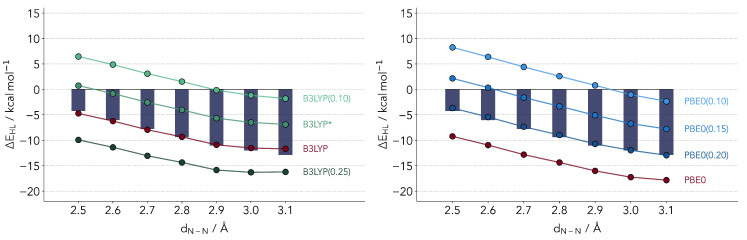
Comparison of Δ*E*_HL_ values computed with refitted B3LYP (**left panel**) and PBE0 (**right panel**) functionals. The bars depict the reference coupled cluster values.

We start by considering common hybrid functionals, which allow for an admixture of Hartree–Fock (exact) exchange according to the general formula

(1) $$E_{\mathrm{xc}}=E_{\mathrm{xc}}^{\mathrm{GGA}}+a\left(E_{x}^{\mathrm{HF}}-E_{x}^{\mathrm{GGA}}\right),$$

whereby a percentage of the GGA exchange energy (PBE in the case of PBE0 [45,46], the Slater–Dirac/B88 exchange energies in the case of B3LYP [47]) is replaced by the Hartree–Fock computed value according to a single parameter *a*. The latter is found to have a major impact on a variety of properties, so much that one can potentially tune the DFT functional to a specific system. Both functionals featured have relatively similar percentages of admixture, with a=0.2 for B3LYP and 0.25 for PBE0. The results for $\Delta E_{\mathrm{HL}}$ with varying *a* are provided in Figure 3. We will denote a functional with a non-default admixture parameter, placing in parenthesis the value of the latter. The B3LYP(0.15) variant, which has been popularized by Reiher and coworkers [48] is denoted as B3LYP*, in line with the common literature nomenclature.

The two profiles for B3LYP and PBE0 look rather similar, both revealing an optimal admixture at 20% exchange. This observation is surprising in several senses. First, it is not too common to observe the same optimal range of admixture for two different functionals. Secondly, at least for B3LYP one would expect a lower optimal value. An admixture of 15% (B3LYP*) has been suggested for the energetics of Fe-S complexes [48]. The latter parameterization has also been validated for the first transition metal row [49]. Still, the overestimation of the LS state is quite visible. We find an almost perfect linear relationship between $\Delta E_{\mathrm{HL}}$ and the value of *a*, a relation which has been hinted upon by several authors but not confirmed for a fixed functional form [24,29,50].

Critically comparing the two functionals, the PBE0(0.20) variant slightly outperforms B3LYP. The reason being that the slope is best reproduced by the parameterized PBE0. B3LYP underestimates the relative stability of HS state by larger distances. This cannot be corrected by changing the parameter *a*. For smaller values the slope will improve but the absolute values will diverge.

#### 2.2.2. Range-Separated Hybrid Functionals

In the case of range-separated hybrid functionals, the two-electron operator for the exchange calculations is split according to the general formula [51,52]

(2) $$r_{12}^{-1}=\frac{1-\left[\alpha +\beta \mathrm{erf}(\mu r_{12})\right]}{r_{12}}+\frac{\alpha +\beta \mathrm{erf}(\mu r_{12})}{r_{12}},$$

whereby α, β and μ are adjustable parameters. The first term on the right stands for the short-range regime, the second for the long range. At a zero interelectronic distance the admixture of exact exchange is given by α. At larger distances, the value will be α+β. For this study, we picked the CAM-B3LYP [52] functional, which in its original form sets α=0.19, β=0.46 and μ=0.33. The underlying functional is the B3LYP hybrid. This class of functionals has been primarily developed for the calculation of electronically excited states and spectra, and are regularly applied in the calculation of spin energetics [20,28,53].

**Figure 4:**
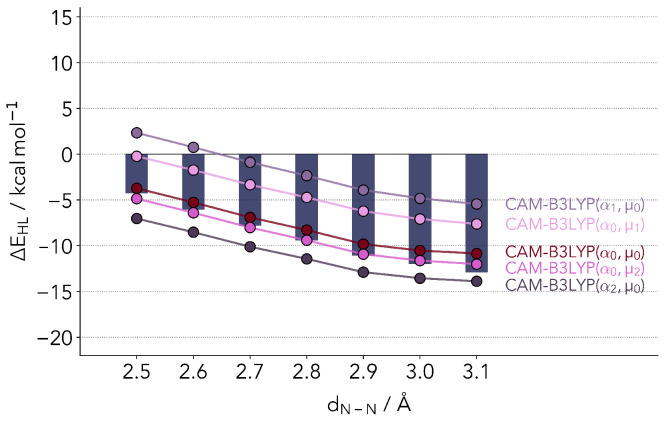
Comparison of Δ*E*_HL_ values computed with refitted CAM-B3LYP. The bars depict the reference coupled cluster values. $\alpha_{0},\mu_{0}$ are the original parameters. $\alpha_{1}$ = 0.13, $\alpha_{2}$ = 0.25, $\mu_{1}$ = 0.25, $\mu_{2}$ = 0.40.

Figure 4 shows the values resulting from calculations with the original set of parameters ($\alpha_{0},\mu_{0}$), as well as slightly lower ($\alpha_{1}=0.13,\mu_{1}=0.25$) and slightly higher ($\alpha_{2}=0.25,\mu_{2}=0.40$) parameters. Just as in the case of B3LYP, the original CAM-B3LYP functional closely reproduces the coupled cluster reference values. The largest differences are observed again for distances above 2.9 Å, with the HS state being too unstable. With different variations of the two parameters we were, however, not able to correct the biggest fault of the parent hybrid functional, the difference in the slope at these larger distances, while maintaining a good accuracy for the absolute $\Delta E_{\mathrm{HL}}$ values.

#### 2.2.3. Local Hybrid Functionals

The underlying idea of the local hybrid PBE0r functional is, first, to formulate the Fock term in a basis of local orbitals $|\chi_{\alpha }\rangle$, and, secondly, to implement the range separation by truncating the sum over four-center integrals, rather than by dividing the Coulomb interaction into short- and long-range contributions. We start by dividing the local orbitals $|\chi_{\alpha }\rangle$ into sets $\alpha \in C_{R}$ that are centered at a specific atom identified by the index *R*. The orbital index α is a combined index holding atomic site, angular momenta and spin indices as well as additional quantum numbers.

The exchange term in the local approximation [54,55] is then given by

(3) $$\begin{array}{c} E_{x}^{\mathrm{PBE}0r}=-\frac{1}{2}\sum_{R}\sum_{\alpha ,\beta ,\gamma ,\delta \in C_{R}}\langle \alpha \beta |\gamma \delta \rangle \rho_{\gamma \beta }^{\left(1\right)}\rho_{\delta ,\alpha }^{\left(1\right)} \end{array}$$

where 〈αβ|γδ〉 stands for 4-center, 2-electron integrals. The one-particle reduced density matrix is given by the occupations $f_{n}$ and Kohn-Sham wave functions $|\psi_{n}\rangle$

(4) $$\begin{array}{ccc} \rho^{\left(1\right)}\left(\alpha ,\beta \right) & = & \sum_{n}\langle \pi_{\alpha }|\psi_{n}\rangle f_{n}\langle \psi_{n}|\pi_{\beta }\rangle \end{array}$$

as well as the local orbital projector functions $\langle \pi_{\alpha }|$, which extract the weight of a local orbital in a Kohn-Sham wave function, i.e.,

(5) $$|\psi_{n}\rangle \approx \sum_{\alpha }|\chi_{\alpha }\rangle \langle \pi_{\alpha }|\psi_{n}\rangle$$

The projector functions obey the bi-orthogonality condition $\langle \pi_{\alpha }|\chi_{\beta }\rangle =\delta_{\alpha ,\beta }$. The approximate sign becomes an identity, if local orbitals span at least the same Hilbert space as the Kohn-Sham wave functions. Equation (3) is an approximation of the exact exchange due to the limitation of the four-center terms to quadruples that are centered on the same atom *R*, i.e., $\{|\chi_{\alpha }\rangle ;\alpha \in C_{R}\}$. This breaks up the Coulomb interaction into atomic contributions.

To avoid the double counting of the exchange term, the pendant of the exchange term in DFT needs to be subtracted. The underlying idea [54] of the double counting term of the PBE0r is to divide up the Coulomb interaction into the contribution of individual atoms using cutoff functions $g_{R}\left(\vec{r}\right)$. Specifically, we partition the electron density into local contributions

(6) $$\begin{array}{c} n_{R}\left(\vec{r}\right)=\sum_{\alpha ,\beta \in C_{R}}\sum_{\sigma }\langle \vec{r},\sigma |\chi_{\alpha }\rangle \rho_{\alpha ,\beta }^{\left(1\right)}\langle \chi_{\beta }|\vec{r},\sigma \rangle \end{array}$$

and define the cutoff functions as $g_{R}\left(\vec{r}\right)=n_{R}\left(\vec{r}\right)/\sum_{R^{\prime }}n_{R^{\prime }}\left(\vec{r}\right)$.

In practice, we simplify the double counting correction by evaluating both cutoff functions at the same position $\vec{r}$. This yields the simple expression

(7) $$\begin{array}{c} E_{\mathrm{DC},\mathrm{approx}}^{\mathrm{PBE}0r}=-\sum_{R}\int d^{3}r\;\frac{n_{R}\left(\vec{r}\right)}{n(\vec{r})}n_{R}\left(\vec{r}\right)\epsilon_{xc}\left(\vec{r}\right) \end{array}$$

Within the PAW code, the density $n(\vec{r})$ is expressed in terms of partial wave expansion, while $n_{R}\left(\vec{r}\right)$ is represented by local orbitals.

This PBE0r method is similar in spirit to the LDA+U method [56]. In contrast to the LDA + U method however, all orbitals on a given site, including core states, are considered, while in the LDA + U approach only a correlated shell such as the d-electrons of a specific atom are taken into account. Furthermore, the double counting term of the PBE0r method differs from the ones used in LDA + U.

Like other range-separated hybrid functionals, there is a close analogy to the GW method [57], an approach based on Green’s function perturbation theory, which has been very successful for describing spectral properties of materials. This is the underlying reason for the improvements of spectral properties in the transition from gradient corrected functionals to hybrid functionals. The range separation of hybrid functionals translates into screening of the long-ranged interaction by the relative dielectric constant of the material. Contrary to CAM-B3LYP, the long-range exact exchange in PBE0r is screened away, in the spirit of the Random-Phase approximation.

PBE0r achieves the range separation by excluding offsite four-center integrals from the exchange and the corresponding double counting term. This provides a reasonable description of the atomic physics, specifically the atomic self-interaction correction. It is thus useful for transition metal compounds with partially filled d-shells. By dividing the hybrid terms into atomic contribution it allows optimization of the admixture of the exact exchange atom by atom. This is the starting point for our next series of parameterizations, whereby the exact exchange percentage is changed according to the atom species. This leads to a 3-dimensional optimization problem, which we tackled with an automated procedure.

**Figure 5:**
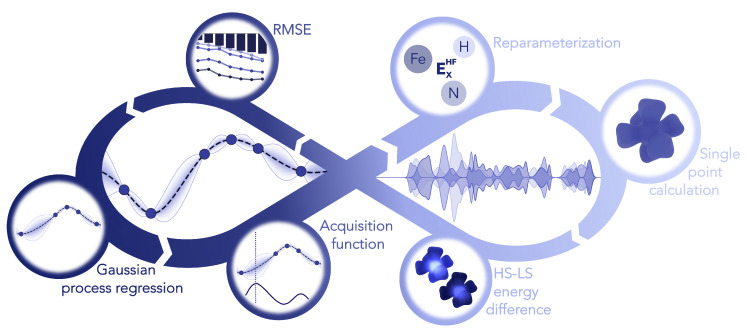
Diagram with an overview of the automated Bayesian optimization of PBE0r HF-exchange weights with the target function being the RMSE relative to the coupled cluster $\Delta E_{\mathrm{HL}}$ values. The process starts with a sampling of different parameter values, computing the DFT $\Delta E_{\mathrm{HL}}$ for the benchmark set. After computing the RMSE a Gaussian process regression is used to provide an acquisition function, selecting new values for the reparameterization and repeating the process.

**Figure 6:**
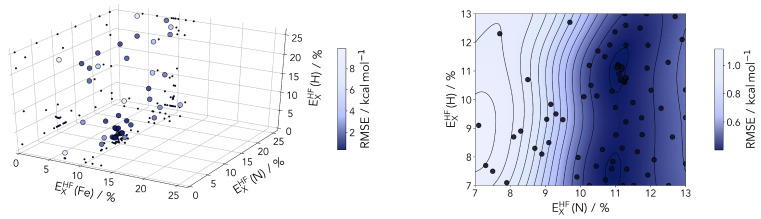
(**Left panel**) sampled points during Bayesian optimization of the HF-exchange percentages for the three atom types. The points are color coded according to the root mean-square error (RMSE) between the computed to the coupled cluster reference. (**Right panel**) 2D-cut of the posterior distribution (at a fixed 9% HF-exchange for Fe) after 80 sampled points. A valley around 11% is clearly identifiable for the N atom scaling.

With the aim of finding the ideal amount of Hartree–Fock exchange for iron, nitrogen and hydrogen we employed Bayesian optimization (see Figure 5). This machine learning tool offers the opportunity to efficiently find the global minimum of a reference by varying a set of parameters [58,59]. Tailored to our case the reference for the optimization is the root-mean-square error (RMSE) between the UCCSD(T*)-F12B $\Delta E_{\mathrm{HL}}$ values and the reparametrized PBE0r functional. The parameters are the element specific Hartree–Fock exchange amounts in percentage. The Bayesian optimization is performed with a Gaussian process regression using a Matérn52 kernel and an expected improvement acquisition function implemented in GPyOpt [60]. During the optimization the Hartree–Fock exchange is varied in the range of 1–25%. For an effective optimization it is crucial to provide a well sampled space to minimize the uncertainty. Therefore, prior to the optimization the whole space is explored with 50 points determined by Latin hypercube sampling [61]. Afterwards additional 50 points are iteratively chosen based on the acquisition function, which is illustrated in Figure 6.

The Bayesian optimization clearly identifies 9% as an ideal value for the HF-exchange by the Fe center. This is found in a range consistent with several previous studies on various classes of transition metal compounds and materials. This includes a recent study on lithium manganese oxides (with the final value for Mn in this particular case also at 9%) [62] and DFT studies of perovskites [34,63].

The HF correction of PBE0r is most important for transition metal ions with partially filled d-shells such as Fe. The influence of the hydrogen parameterization should always be marginal, and this is clear in the posterior distribution plotted in Figure 6 Nonetheless, considering the direct coordination to the metal, the description of the nitrogen centers should be looked upon with some care, even if the first results hint at a small influence on the RMSE.

**Figure 7:**
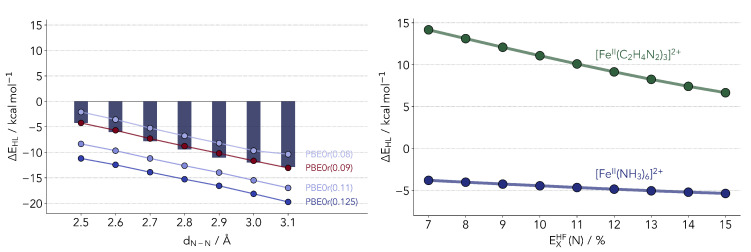
(**Left panel**) Comparison of Δ*E*_HL_ values computed with PBE0r. The numbers indicate the amount of exact exchange for all atom types. The bars depict the reference coupled cluster values. (**Right panel**) Comparison of the dependence of the Δ*E*_HL_ on the exchange admixture.

To observe if this (lack of) dependence on the nitrogen HF-exchange fraction is kept when considering more realistic models of SCO complexes, we devised a second model with ethane-1,2-diimine (with the chemical formula C_2_H_4_N_2_) as ligand. The latter is a small bidentate ligand molecule which will more closely mimic SCO complexes. In a new series of calculations, we varied the percentage of local exchange at the nitrogen atoms while keeping every other center at 9%. The results are shown in Figure 7. Although we do not provide reference data for the diimine model [Fe^II^(C_2_H_4_N_2_)_3_]^2+^, one can straightforwardly compare the dependence of $\Delta E_{\mathrm{HL}}$ on the exchange admixture. It is visible in Figure 7 that the dependence is larger than in our simplest model system. Changes in the exchange functional for the local density located at the nitrogen atoms will impact the charge distribution along the double bond to the carbon, in turn affecting the coordination to the metal. In the simpler hexaamino ion, one only had protons which take up little density.

## 3. Discussion

The evidence presented from our wave function analysis strongly hints at the single-reference character of both singlet and quintet states in the [Fe^II^(NH_3_)_6_]^2+^ model. This further supports coupled cluster as an adequate reference method in the discussion of spin-state energetics for these systems. We were able to confirm for the most part the local coupled cluster results of Flöser et al. [33], but took a different approach in the benchmarking of DFT functionals. The changes in coordination while keeping the same ligands provides insight into how the DFT method performs when structural changes occur in the complex, a fundamental issue in the theoretical description of SCO complexes.

The results show that although many functionals can be tuned to replicate a reference singlet-quintet gap $\Delta E_{\mathrm{HL}}$, the trends upon structural changes are much harder to capture. It is also observed that only the GGA functionals or hybrids with starkly reduced percentages of non-local exchange obtain an agreement with the coupled cluster $\Delta E_{\mathrm{HL}}$ slope (energy gap as a function of the ligand structure). This observation certainly validates the approach of Jakubikova [9], bearing in mind that the model system is quite small.

The best performing (parameterized) functionals were the hybrid PBE0(0.20) and the local hybrid PBE0r with an admixture of 0.09 at the Fe center. The latter has a much-reduced computational cost and could be readily employed for the simulation of larger systems, even oligo-nuclear SCO complexes. This is current work in progress in our labs. However, the results also raise some red flags. As it is visible in Figure 7, the hexaamino complex may only tell part of the story. One could increase the chemical diversity with other model systems, such as [Fe(H_2_O)_6_]^2+^, [Fe(NCH)_6_]^2+^) but these will not suffice. They lack some very important factors weighing on common SCO complexes, such as the coordination to conjugated systems or the distortion of the octahedral geometry (due to ligand constraints and steric effects). Our model distortions provide a proof of principle but only scratch the surface. The way forward might warrant the inclusion of more realistic models and be more demanding regarding the multi-dimensional tuning of the local hybrid functional. Imine-based ligands would be a clear priority in this respect, given the ease in their synthesis and the widespread use in tailored Fe(II) SCO complexes. One would also have to consider coordination to oxygen (for a recent example of ligands combining imine and alcohol coordinating moieties, see Ref. [64]), and that alone brings the number of elements to a total of four, disregarding the hydrogens.

The automated parameterization procedure here applied should effectively deal with increased dimensionality, when moving to more realistic ligands involving other atom types. In fact, the [Fe^II^(NH_3_)_6_]^2+^ parameterization was rather trivial and there is room for added challenges. The question lies more in obtaining the needed reference data. On the basis of coupled cluster, if static correlation is kept at bay, explicit correlated local wave function methods [65] could be applied to extend the study range while sacrificing very little in terms of accuracy.

## 4. Materials and Methods

### 4.1. Coupled Cluster Calculations

To reach almost complete basis set limit accuracy and to keep the computational costs as manageable as possible, explicit correlation methods have been employed [66]. The reference results for the benchmark have been obtained with the unrestricted coupled cluster F12B method [67] including single, double and perturbative triple excitations with the fixed amplitude 3C(FIX) ansatz [68] implemented in MOLPRO [69]. Additional complementary auxiliary basis set (CABS) singles correction and scaling of the perturbative triples were applied [70]. For the ammonia ligands the method specific cc-pVTZ-F12 basis sets was used [71]. For iron, the aug-cc-pwCVTZ basis was chosen [72], which allows for an adequate recovery of the 3s3p correlation. Following the prior work on explicit correlation methods on transition metal complexes from Bross et al. [73], we used their aug-cc-pwCVTZ/MP2FIT as the density fitting basis and as the CABS for the resolution of the identity. For the density fitting of the Fock and exchange matrices def2-QZVPP [74] was used. The Hartree–Fock calculations, for the recovery of the relativistic contributions to the F12B energy, were carried out with a cc-pwCVQZ-DK/cc-pwCVQZ [72] basis for iron and cc-pVQZ-DK/cc-pVQZ [75] for the remainder.

### 4.2. Density Functional Calculations

The ORCA 4.2.0 program package [76,77] was employed for all DFT calculations except the local hybrid functional PBE0r. The range of functionals in these single point energy calculations included the GGAs PBE [35] and BP86 [36,37], the meta-GGAs TPSS [38] and M06-L [39], the hybrid GGAs PBE0 [45,46] and B3LYP [47] (both with varying amounts of exact exchange), and the range-separated hybrid functional CAM-B3LYP [52] (with variation in the amount of initial exact exchange and in the distance parameter). The cc-pwCVQZ-DK [72] basis set was used for the Fe atom and cc-pVQZ-DK [75] for the remaining atoms. Relativistic effects are taken into account by using the second-order Douglas-Kroll-Hess Hamiltonian (DKH2) [78,79]. The RIJCOSX approximation [80] was used to speed up calculation time. Grimme’s D3 method with Becke–Johnson damping [40,41] was used for dispersion correction. By using the ’verytightscf’ keyword, the convergence threshold was set to $10^{-9}$ H.

The calculations with PBE0r were carried out using the Car–Parinello Projector Augmented-Wave (CP-PAW) code package [81,82]. Initial geometry optimizations were carried out using 12.5% exact exchange for all atom types. An example of a corresponding input file is given in the Supplementary Materials.

## Supplementary Materials

The following are available online, sample input of CP-PAW calculation, structures of the hexaamino model under N-N restraints, absolute DFT energies for the different structures.

## Abbreviations

The following abbreviations are used in this manuscript:

SCO	spin crossover
HS	high spin
LS	low spin
DFT	density functional theory
RASSCF	restricted active space self-consistent field
RASPT2	restricted active space second-order perturbation
CASSCF	complete active space self-consistent field
CASPT2	complete active space second-order perturbation
NEVPT2	N-electron valence state perturbation theory
SORCI	spectroscopy-oriented configuration interaction
CCSD(T)	coupled cluster with single, double and perturbative triple excitations
DMC	diffusion Monte Carlo
GGA	generalized gradient approximation
LDA	local-density approximation
RMSE	root-mean-square error
HF	Hartree–Fock
CBS	complete basis set
CABS	complementary auxiliary basis set
DKH2	Douglas-Kroll-Hess Hamiltonian
CP-PAW	Car–Parinello Projector Augmented-Wave

## Appendix A

As shown in Figure A1 (left panel) the primary trend of the reference energy in dependence of the nitrogen pair distance is mainly given by the CCSD energy for distances shorter than 2.9 Å. However, for larger distances the CCSD energy goes into saturation and the overall trend is given by the decreasing energy contribution of the perturbative triple excitations. Furthermore, the corrections for the perturbative triples and the relativistic effects are largely geometry-independent.

On the right side of Figure A1 the energy difference between a relativistic and non-relativistic canonical CCSD(T) calculations for the Fe(II) hexaamino complex is shown. The computation was performed with a second-order Douglas-Kroll-Hess Hamiltonian (DKH2) [78,79] using a cc-pwCVTZ-DK/cc-pwCVTZ [72] basis set for the iron atom and a cc-pVDZ-DK/cc-pVDZ [75] basis for the ligand atoms. The energy difference shows that the correlation energy is largely independent from relativistic effects, albeit the reference energy is strongly affected. Thus, it is confirmed as a reasonable approximation to recover the relativistic effects solely from the reference calculations.
