# Supplementary material for: A Review of Density Functional Models for the Description of Fe(II) Spin-Crossover Complexes
Source: Molecules. 2020 Nov 6;25(21):5176. doi: 10.3390/molecules25215176 (PMC7664392; doi:10.3390/molecules25215176)
Supplement: Supplementary file 1 [file molecules-25-05176-s001.pdf]

A review of density functional models for the description of Fe(II)-based spin crossover complexes - Supporting Information

Anton Römer[1], Lukas Hasecke[1], Peter Blöchl[2,3] and Ricardo A. Mata[1]*

[1] Institute of Physical Chemistry, University of Goettingen, D-37077 Göttingen, Germany;
[2] Institute of Theoretical Physics, University of Goettingen, D-37077 Göttingen, Germany;
[3] Institute of Theoretical Physics, Clausthal University of Technology, 38678 Clausthal-Zellerfeld, Germany;
* Correspondence: rmata@gwdg.de

1 Example of input file for CP-PAW calculations

```
!STRUCTURE
 !GENERIC LUNIT[AA]=1. !END
 !OCCUPATIONS EMPTY=10 NSPIN=2 SPIN[HBAR]=2. CHARGE[E]=2. !END
 !CONSTRAINTS
    !BOND ATOM1='N_01' ATOM2='N_02' MOVE=T NSTEP=1 VALUE=4.724 !END
    !BOND ATOM1='N_03' ATOM2='N_04' MOVE=T NSTEP=1 VALUE=4.724 !END
    !BOND ATOM1='N_05' ATOM2='N_06' MOVE=T NSTEP=1 VALUE=4.724 !END
 !END
 !SPECIES NAME='FE' NPRO=1 1 1  LRHOX=4
    !NTBO    NOFL=1 0 1 CV=T  raug/rcov=1.15 LHFWEIGHT=0.080
             focksetup=F cv=T NDDO=F 31=F BONDX=F !END
    !AUGMENT  ID='FE_NDLSS_V0' Z=  26.00000 ZV= 8.
             TYPE='NDLSS' RBOX/RCOV=  1.200 RCSM/RCOV=  0.250
             RCL/RCOV=  0.800  0.800  0.800  0.800  0.800
      !GRID DMIN=   0.100E-05 DMAX=     0.100 RMAX=    20.000 !END
      !POT  POW=  3.000 RC/RCOV=  0.702 !END
      !CORE POW=  3.000 RC/RCOV=  0.702 !END
    !END
 !END
 !SPECIES   NAME='N_'   NPRO=1 1 1 LRHOX=4 RAD/RCOV=1.4
    !NTBO    NOFL=1 1 0 CV=T  RAUG/RCOV=1.2 LHFWEIGHT=0.080
             FOCKSETUP=F CV=T NDDO=F 31=F BONDX=F !END
    !AUGMENT ID='MY_NDLSS_N' EL='N' ZV= 5.
             TYPE='NDLSS' RBOX/RCOV=1.2 RCSM/RCOV=.25
             RCL/RCOV=0.75 0.75 0.75  0.75
      !GRID  DMIN=1.E-6 DMAX=.15 RMAX=9. !END
      !POT   POW=3. RC/RCOV=0.75 VAL0_X=-4.3 !END
      !CORE  POW=2. RC/RCOV=0.75  !END
    !END
 !END
 !SPECIES   NAME='C_'   NPRO=1 1 1 LRHOX=4 RAD/RCOV=1.4
    !NTBO    NOFL=1 1 0 CV=T  RAUG/RCOV=1.2 LHFWEIGHT=0.080
             FOCKSETUP=F CV=T NDDO=F 31=F BONDX=F !END
    !AUGMENT ID='MY_NDLSS_C' EL='C' ZV= 4.
             TYPE='NDLSS' RBOX/RCOV=1.2 RCSM/RCOV=.25
             RCL/RCOV=0.85 0.85 0.85 0.85
      !GRID  DMIN=1.E-6 DMAX=.15 RMAX=9. !END
      !POT   POW=3. RC/RCOV=0.75  VAL0_X=-2.7 !END
      !CORE  POW=2. RC/RCOV=0.75  !END
    !END
 !END
 !SPECIES   NAME='H_'  M=2. NPRO=1 1 LRHOX=2 RAD/RCOV=1.2
    !NTBO    NOFL=1 0 CV=T  RAUG/RCOV=1.2 LHFWEIGHT=0.080
             FOCKSETUP=F CV=T NDDO=F 31=F BONDX=F !END
    !AUGMENT ID='MY_NDLSS_H' EL='H' ZV= 1.
             TYPE='NDLSS' RBOX/RCOV=1.2 RCSM/RCOV=.25
             RCL/RCOV=1.2 1.2  1.2  1.2
      !GRID  DMIN=1.E-6 DMAX=.15 RMAX=9. !END
      !POT   POW=3. RC/RCOV=1. VAL0_X=-1.6 !END
      !CORE  POW=2. RC/RCOV=1. !END
    !END
 !END
!LATTICE T=   0.00000 6.90000 6.90000
              6.90000 0.00000 6.90000
              6.90000  6.90000 0.0000 !END
 !ATOM NAME= 'FE1' R=    0.78135    3.03110   12.31354 !END
 !ATOM NAME= 'N_01' R=    -0.38282    3.30934   10.52564 !END
```

```
 !ATOM NAME= 'N_02' R=    -0.54065    1.35312   12.07401 !END
 !ATOM NAME= 'N_03' R=     2.47003    2.14026   11.31866 !END
 !ATOM NAME= 'N_04' R=     2.24845    4.55400   11.93017 !END
 !ATOM NAME= 'N_05' R=     1.39773    2.68495   14.34201 !END
 !ATOM NAME= 'N_06' R=    -0.53750    4.10138   13.63647 !END
 !ATOM NAME= 'H_01' R=    -0.24074    4.17232    9.96608 !END
 !ATOM NAME= 'H_02' R=    -1.37693    4.56787   13.24192 !END
 !ATOM NAME= 'H_03' R=    -0.33027    2.62500    9.74887 !END
 !ATOM NAME= 'H_04' R=    -1.56828    1.49211   12.05918 !END
 !ATOM NAME= 'H_05' R=    -0.51014    0.58817   12.77534 !END
 !ATOM NAME= 'H_06' R=     3.38076    2.12723   11.81349 !END
 !ATOM NAME= 'H_07' R=     2.46407    4.80044   10.94669 !END
 !ATOM NAME= 'H_08' R=     2.06357    5.51685   12.27294 !END
 !ATOM NAME= 'H_09' R=     2.26719    2.14322   14.51056 !END
 !ATOM NAME= 'H_10' R=     2.43216    1.13026   11.08004 !END
 !ATOM NAME= 'H_11' R=     1.59441    3.47852   14.97880 !END
 !ATOM NAME= 'H_12' R=    -0.99859    3.62098   14.43059 !END
 !ATOM NAME= 'H_13' R=    -0.15737    4.92145   14.14454 !END
 !ATOM NAME= 'H_14' R=     0.77476    2.13293   14.95971 !END
 !ATOM NAME= 'H_15' R=     3.21643    4.45779   12.28865 !END
 !ATOM NAME= 'H_16' R=     2.79258    2.49364   10.39885 !END
 !ATOM NAME= 'H_17' R=    -1.41404    3.37299   10.61104 !END
 !ATOM NAME= 'H_18' R=    -0.44779    0.76295   11.22736 !END
 !ISOLATE !END
!END
!EOB
```

2 Cartesian coordinates of the $[Fe^{II}(NH_3)_6]^{2+}$ model complexes

Table 1: N-N pair distance 2.5 Å, high-spin.

FE	0.78135	3.03110	12.31354
N	-0.38282	3.30934	10.52564
N	-0.54065	1.35312	12.07401
N	2.47003	2.14026	11.31866
N	2.24845	4.55400	11.93017
N	1.39773	2.68495	14.34201
N	-0.53750	4.10138	13.63647
H	-0.24074	4.17232	9.96608
H	-1.37693	4.56787	13.24192
H	-0.33027	2.62500	9.74887
H	-1.56828	1.49211	12.05918
H	-0.51014	0.58817	12.77534
H	3.38076	2.12723	11.81349
H	2.46407	4.80044	10.94669
H	2.06357	5.51685	12.27294
H	2.26719	2.14322	14.51056
H	2.43216	1.13026	11.08004
H	1.59441	3.47852	14.97880
H	-0.99859	3.62098	14.43059
H	-0.15737	4.92145	14.14454
H	0.77476	2.13293	14.95971
H	3.21643	4.45779	12.28865
H	2.79258	2.49364	10.39885
H	-1.41404	3.37299	10.61104
H	-0.44779	0.76295	11.22736

Table 2: N-N pair distance 2.5 Å, low-spin.

FE	0.84908	3.00986	12.33825
N	-0.21820	3.18064	10.70853
N	-0.23274	1.38088	12.44341
N	2.30667	2.18910	11.32266
N	1.96856	4.57742	11.97896
N	1.69689	2.78426	14.08995
N	-0.43064	3.94914	13.48509
H	-0.13751	4.03582	10.12640
H	-1.31644	4.31314	13.08462
H	-0.11354	2.47812	9.95265
H	-1.26398	1.46192	12.52459
H	-0.07841	0.70284	13.21368
H	3.26584	2.24186	11.71256
H	2.20086	4.80525	10.99486
H	1.64486	5.52030	12.26859
H	2.61013	2.29724	14.16794
H	2.29129	1.17003	11.12574
H	1.92920	3.62675	14.64836
H	-0.83198	3.45280	14.30257
H	-0.12653	4.81892	13.96062
H	1.19073	2.24647	14.81745
H	2.92325	4.61991	12.38267
H	2.50588	2.52244	10.36074
H	-1.25129	3.16694	10.79513
H	-0.18743	0.70986	11.65492

Table 3: N-N pair distance 2.6 Å, high-spin.

FE	0.80639	2.99890	12.27428
N	-0.23931	3.27765	10.40205
N	-0.53974	1.32249	12.08914
N	2.51466	2.04084	11.35952
N	2.26294	4.56074	11.94770
N	1.40233	2.64449	14.32132
N	-0.55356	4.14815	13.50113
H	-0.04143	4.12676	9.83998
H	-1.36587	4.60866	13.04915
H	-0.15357	2.57508	9.64467
H	-1.56361	1.48181	12.04722
H	-0.53342	0.59136	12.82482
H	3.41654	2.05628	11.87026
H	2.58720	4.74733	10.98122
H	2.01675	5.53517	12.20576
H	2.25595	2.08477	14.50548
H	2.47507	1.02040	11.17553
H	1.60454	3.44122	14.95264
H	-1.05466	3.70797	14.29464
H	-0.17911	4.98077	13.99245
H	0.75428	2.11684	14.93438
H	3.18587	4.51233	12.41769
H	2.85498	2.34630	10.42889
H	-1.27246	3.36108	10.42278
H	-0.44308	0.69689	11.26883

Table 4: N-N pair distance 2.6 Å, low-spin.

FE	0.70250	2.87900	12.33632
N	-0.21489	2.90053	10.59577
N	-0.37848	1.25177	12.59927
N	2.20918	1.89539	11.54440
N	1.81848	4.44170	11.89491
N	1.46814	2.82949	14.15056
N	-0.68469	3.95787	13.22791
H	-0.06096	3.69293	9.94483
H	-1.52133	4.26315	12.69595
H	-0.05517	2.12281	9.92882
H	-1.41257	1.33234	12.61577
H	-0.25822	0.68530	13.45964
H	3.14620	2.00380	11.97455
H	2.18942	4.52753	10.93087
H	1.42115	5.39567	11.98495
H	2.36198	2.33098	14.31965
H	2.18401	0.85881	11.50989
H	1.70165	3.72439	14.61998
H	-1.16861	3.56790	14.05805
H	-0.41769	4.87930	13.62101
H	0.91412	2.39477	14.91127
H	2.70174	4.59532	12.41644
H	2.46936	2.07328	10.55620
H	-1.25177	2.90353	10.58571
H	-0.29206	0.48555	11.90690

Table 5: N-N pair distance 2.7 Å, high-spin.

FE	8.11007	6.62689	5.90026
N	6.92941	7.01594	7.68232
N	7.63022	4.63339	6.62328
N	6.38862	6.81931	4.58397
N	8.19922	8.70264	5.26519
N	10.06174	6.64575	6.87158
N	9.46016	5.97546	4.32638
H	9.08598	5.80548	3.37462
H	8.96258	9.31110	5.61376
H	10.27311	6.56512	4.06978
H	10.62989	5.77987	6.91594
H	10.11641	6.91487	7.87168
H	6.56070	7.03057	3.58366
H	7.40091	9.32032	5.49929
H	7.96977	3.81216	6.08952
H	6.69739	7.99719	7.92330
H	5.76941	5.99836	4.45195
H	6.63325	4.35868	6.69149
H	7.31885	6.73041	8.59944
H	5.98199	6.60614	7.77644
H	7.94061	4.34047	7.56814
H	5.65852	7.53117	4.76997
H	8.27710	8.92486	4.25539
H	10.78769	7.29187	6.51160
H	9.95441	5.07323	4.45165

Table 6: N-N pair distance 2.7 Å, low-spin.

FE	8.09650	6.59150	5.88097
N	7.23944	7.21542	7.55215
N	7.76871	4.76060	6.56071
N	6.34659	6.62208	4.94831
N	8.31422	8.45012	5.22395
N	9.88679	6.60122	6.73455
N	9.02845	5.91160	4.26940
H	8.49269	5.73870	3.39902
H	9.16648	8.98690	5.46919
H	9.79952	6.47542	3.86665
H	10.38786	5.70282	6.86360
H	9.98714	6.97484	7.69652
H	6.33646	6.77584	3.92305
H	7.60668	9.15700	5.49524
H	7.84164	3.95505	5.91253
H	7.45128	8.16955	7.89866
H	5.73717	5.78426	4.98081
H	6.83680	4.54886	6.96256
H	7.42305	6.69179	8.42767
H	6.20488	7.25791	7.61168
H	8.35711	4.39261	7.33120
H	5.64367	7.33520	5.21662
H	8.31629	8.61775	4.20069
H	10.65000	7.13878	6.28412
H	9.51788	5.00000	4.32933

Table 7: N-N pair distance 2.8 Å, high-spin.

FE	8.08091	6.59883	5.92752
N	6.87245	7.05523	7.68722
N	7.59348	4.58055	6.59388
N	6.35472	6.74433	4.58644
N	8.23774	8.68549	5.31130
N	9.98238	6.60132	7.01563
N	9.43954	5.96423	4.34379
H	9.08518	5.84944	3.37676
H	8.99305	9.28127	5.69645
H	10.27848	6.53680	4.13751
H	10.58247	5.75626	7.01165
H	9.96775	6.77533	8.03774
H	6.53006	6.96717	3.58923
H	7.43477	9.30882	5.51121
H	7.85946	3.78232	5.98899
H	6.81880	8.04092	8.00268
H	5.76103	5.90662	4.44502
H	6.59738	4.33374	6.73825
H	7.12517	6.62136	8.59369
H	5.85900	6.83663	7.68396
H	7.97544	4.23029	7.49157
H	5.60408	7.43558	4.76846
H	8.36168	8.90956	4.30665
H	10.70046	7.30429	6.76227
H	9.90110	5.04198	4.44494

Table 8: N-N pair distance 2.8 Å, low-spin.

FE	8.09750	6.58190	5.91287
N	7.18000	7.26004	7.54556
N	7.73184	4.71502	6.51715
N	6.38601	6.56397	4.89131
N	8.39805	8.46279	5.32196
N	9.82926	6.56698	6.90882
N	9.05923	5.91425	4.29725
H	8.55387	5.85680	3.39443
H	9.07353	9.05502	5.83905
H	9.89780	6.43188	3.97626
H	10.44895	5.74279	6.80722
H	9.81274	6.62291	7.94390
H	6.25570	7.24736	4.12326
H	7.58954	9.11085	5.32711
H	7.75340	3.94409	5.82510
H	7.51008	8.14960	7.96287
H	6.13359	5.70051	4.37644
H	6.81011	4.51285	6.94590
H	7.18780	6.67415	8.40041
H	6.16598	7.47316	7.51355
H	8.33846	4.29046	7.24237
H	5.48916	6.71398	5.38856
H	8.73906	8.64390	4.35993
H	10.52842	7.30947	6.72566
H	9.46460	4.96048	4.31064

Table 9: N-N pair distance 2.9 Å, high-spin.

FE	1.67617	6.62173	-0.33134
N	2.23256	4.50201	-0.23771
N	3.71924	6.89992	0.43249
N	0.83583	6.53656	1.69445
N	-0.39280	6.13794	-0.90188
N	1.34788	8.77909	-0.48142
N	2.30636	6.84193	-2.41484
H	1.43692	6.73284	2.51523
H	3.16628	7.37465	-2.63981
H	0.61237	9.15046	-1.10973
H	1.08734	9.28470	0.38445
H	0.40386	5.65427	2.02494
H	-1.14278	6.84119	-0.77141
H	-0.59448	5.87640	-1.88462
H	3.99945	7.83292	0.78697
H	1.56385	3.77787	-0.55688
H	4.01179	6.33613	1.25148
H	3.05945	4.20610	-0.78751
H	2.51089	5.97747	-2.94787
H	-0.85366	5.33385	-0.43794
H	0.05526	7.18199	1.91240
H	2.13472	9.38336	-0.78009
H	1.65924	7.28836	-3.08969
H	4.53417	6.71526	-0.18061
H	2.49357	4.08256	0.67332

Table 10: N-N pair distance 2.9 Å, low-spin.

FE	1.70831	6.57455	-0.33572
N	1.96093	4.58669	-0.37424
N	3.58304	6.89667	0.29069
N	1.13100	6.43581	1.57700
N	-0.16317	6.32122	-1.01557
N	1.38403	8.55165	-0.26413
N	2.35764	6.65496	-2.22984
H	1.70596	5.85579	2.21485
H	2.56301	7.57467	-2.66128
H	1.42298	9.10763	-1.13777
H	0.46642	8.87983	0.08840
H	0.20068	6.03569	1.79823
H	-0.96133	6.38945	-0.35845
H	-0.50241	6.97498	-1.74434
H	3.73332	7.30291	1.23228
H	1.87992	4.08914	-1.27962
H	4.24569	6.10285	0.35573
H	2.85942	4.18006	-0.05545
H	3.24735	6.17380	-2.45542
H	-0.41514	5.42655	-1.47398
H	1.07213	7.29489	2.15336
H	2.00079	9.14258	0.32285
H	1.76719	6.25995	-2.98437
H	4.17294	7.54802	-0.25837
H	1.31608	4.00057	0.18641

Table 11: N-N pair distance 3.0 Å, high-spin.

FE	1.65567	6.63817	-0.34177
N	2.11809	4.49235	-0.15053
N	3.67079	6.97655	0.49547
N	0.76899	6.61274	1.67726
N	-0.38187	6.17608	-1.05848
N	1.31852	8.80214	-0.53927
N	2.42272	6.72262	-2.39824
H	1.36836	6.77848	2.50582
H	3.24523	7.31423	-2.61521
H	0.63536	9.15620	-1.23316
H	0.97790	9.31056	0.29632
H	0.27863	5.76028	2.00428
H	-1.10951	6.91348	-1.04450
H	-0.51035	5.84514	-2.03219
H	3.89555	7.90231	0.90367
H	1.45877	3.79380	-0.53859
H	3.97002	6.38421	1.29159
H	2.99072	4.15091	-0.59281
H	2.75112	5.83379	-2.81712
H	-0.91324	5.42878	-0.57540
H	0.02485	7.30487	1.87801
H	2.12025	9.41418	-0.77621
H	1.79563	7.03224	-3.16261
H	4.50772	6.86109	-0.10426
H	2.24984	4.07815	0.79018

Table 12: N-N pair distance 3.0 Å, low-spin.

FE	1.73145	6.56618	-0.33890
N	1.99308	4.56064	-0.39675
N	3.59994	6.99248	0.31288
N	1.18242	6.40375	1.60050
N	-0.12183	6.27105	-1.09779
N	1.31853	8.54246	-0.21217
N	2.41692	6.62553	-2.24155
H	1.74895	5.78098	2.20403
H	2.61733	7.54087	-2.68409
H	1.34616	9.12799	-1.06651
H	0.37948	8.81043	0.13377
H	0.24110	6.03790	1.83373
H	-0.94455	6.28908	-0.46849
H	-0.45207	6.94410	-1.81252
H	3.71829	7.44479	1.23737
H	1.89883	4.06778	-1.30306
H	4.29084	6.22833	0.42216
H	2.89650	4.15382	-0.09267
H	3.31863	6.15376	-2.43551
H	-0.32720	5.38720	-1.59769
H	1.17459	7.24847	2.19987
H	1.89669	9.14096	0.40494
H	1.84729	6.20885	-2.99998
H	4.16954	7.64096	-0.25971
H	1.35563	3.97600	0.17315

Table 13: N-N pair distance 3.1 Å, high-spin.

FE	1.67865	6.57736	-0.37345
N	2.04971	4.38831	-0.37834
N	3.66827	6.88660	0.48672
N	0.87690	6.45765	1.66167
N	-0.32198	6.23306	-1.18819
N	1.31621	8.76746	-0.42901
N	2.48935	6.70392	-2.42275
H	1.23321	5.72775	2.30498
H	2.89857	7.60535	-2.72765
H	0.82223	9.15545	-1.25340
H	0.73853	9.20382	0.31233
H	-0.13757	6.28097	1.77311
H	-1.13118	6.29156	-0.54539
H	-0.67677	6.85228	-1.93966
H	3.86472	7.76608	0.99795
H	1.89138	3.86781	-1.26060
H	4.02935	6.21279	1.18605
H	2.99910	4.03238	-0.16378
H	3.27588	6.07716	-2.67103
H	-0.53258	5.31757	-1.62535
H	0.96355	7.27689	2.28995
H	2.11950	9.42150	-0.41096
H	1.88019	6.52290	-3.24073
H	4.47664	6.89398	-0.16031
H	1.50450	3.77854	0.25793

Table 14: N-N pair distance 3.1 Å, low-spin.

FE	1.74681	6.59135	-0.38339
N	1.86318	4.55940	-0.56911
N	3.62144	6.93933	0.35497
N	1.17047	6.31638	1.55526
N	-0.08997	6.45963	-1.27322
N	1.39164	8.58413	-0.09661
N	2.52523	6.68398	-2.26776
H	1.72296	5.64145	2.11353
H	2.80957	7.60047	-2.65881
H	1.43027	9.23270	-0.90336
H	0.45994	8.84295	0.27438
H	0.22094	5.95524	1.75976
H	-0.94613	6.47433	-0.69044
H	-0.33709	7.20905	-1.94357
H	3.72192	7.33074	1.30869
H	1.77961	4.13276	-1.50915
H	4.29750	6.15827	0.43189
H	2.71302	4.06186	-0.24708
H	3.40127	6.15776	-2.43535
H	-0.31134	5.63271	-1.85611
H	1.17662	7.11908	2.20933
H	1.98806	9.11718	0.56129
H	1.96602	6.34038	-3.06893
H	4.21304	7.61251	-0.16394
H	1.14944	4.00009	-0.06900

3 Absolute energies from the SPE calculations on the $[Fe^{II}(NH_3)_6]^{2+}$ model complexes

3.1 Using PBE, BP86, TPSS and M06-L

	PBE	BP86	TPSS	M06-L
2.5 Å, HS	-1611.31394182704	-1612.21181225286	-1612.04541650429	-1611.8312296385
2.5 Å, LS	-1611.33504264393	-1612.23203305343	-1612.06943423148	-1611.82617037154
2.6 Å, HS	-1611.32669581554	-1612.22458186504	-1612.05819837707	-1611.84543307343
2.6 Å, LS	-1611.34489446973	-1612.24198183326	-1612.0794918585	-1611.83691670181
2.7 Å, HS	-1611.33581983985	-1612.23372142758	-1612.06728580552	-1611.85489181217
2.7 Å, LS	-1611.35084864319	-1612.248039278	-1612.08563838146	-1611.84367729907
2.8 Å, HS	-1611.34174799896	-1612.23970247011	-1612.07307793425	-1611.86089127433
2.8 Å, LS	-1611.35387535836	-1612.25111882385	-1612.08887837626	-1611.84808257695
2.9 Å, HS	-1611.34546305524	-1612.2434864492	-1612.07701036119	-1611.86492073313
2.9 Å, LS	-1611.35464639159	-1612.25190109507	-1612.08984512315	-1611.85059770242
3.0 Å, HS	-1611.34789125528	-1612.24553346853	-1612.07906433559	-1611.86717974695
3.0 Å, LS	-1611.35434760779	-1612.25164217608	-1612.0897802799	-1611.85213183204
3.1 Å, HS	-1611.34703690399	-1612.24497736748	-1612.07845143695	-1611.86621919722
3.1 Å, LS	-1611.35214635849	-1612.2494748855	-1612.08773294594	-1611.85178194351

3.2 Using B3LYP with 10, 15, 20 and 25% exact exchange

	B3LYP(10%)	B3LYP(15)	B3LYP(20)	B3LYP(25%)
2.5 Å, HS	-1610.94952594267	-1611.33284328771	-1611.71736748635	-1612.10304650325
2.5 Å, LS	-1610.95982682662	-1611.33402051468	-1611.70983687101	-1612.08721943538
2.6 Å, HS	-1610.96271412583	-1611.34636099819	-1611.73120044291	-1612.11718169432
2.6 Å, LS	-1610.97047172445	-1611.34506518765	-1611.72127420517	-1612.09904226486
2.7 Å, HS	-1610.97217288164	-1611.35610715504	-1611.74122273941	-1612.12747017399
2.7 Å, LS	-1610.97709332561	-1611.35202710027	-1611.72857203556	-1612.1066711232
2.8 Å, HS	-1610.97826791859	-1611.3624037942	-1611.74773319818	-1612.13415231224
2.8 Å, LS	-1610.98071558478	-1611.35597638502	-1611.73284655096	-1612.11126838357
2.9 Å, HS	-1610.98218728739	-1611.36649115501	-1611.75196277805	-1612.13855274433
2.9 Å, LS	-1610.98194828978	-1611.35747163195	-1611.73460230896	-1612.11328218151
3.0 Å, HS	-1610.98431129668	-1611.36873438923	-1611.75432275792	-1612.14102661488
3.0 Å, LS	-1610.98243579007	-1611.35838715145	-1611.7359427516	-1612.11504346795
3.1 Å, HS	-1610.98381943511	-1611.36825944887	-1611.75385919713	-1612.14057173513
3.1 Å, LS	-1610.98093853655	-1611.35728324445	-1611.73523147549	-1612.11472270883

3.3 Using PBE0 with 10, 15, 20 and 25% exact exchange

	PBE0(10%)	PBE0(15%)	PBE0(20%)	PBE0(25%)
2.5 Å, HS	-1610.19032949751	-1610.57443830335	-1610.95974208347	-1611.34619005557
2.5 Å, LS	-1610.2034804874	-1610.57788548789	-1610.95390384975	-1611.33148349425
2.6 Å, HS	-1610.20265934361	-1610.58710726414	-1610.97273611442	-1611.35949646403
2.6 Å, LS	-1610.2128425653	-1610.58765273006	-1610.96407110037	-1611.34204471464
2.7 Å, HS	-1610.21125206993	-1610.59599630844	-1610.98191177494	-1611.36894841734
2.7 Å, LS	-1610.21828956788	-1610.59344298127	-1610.97020205597	-1611.34851340839
2.8 Å, HS	-1610.21681329215	-1610.60171221025	-1610.98781482125	-1611.37504807853
2.8 Å, LS	-1610.22093900877	-1610.59642377858	-1610.97351412287	-1611.35215618729
2.9 Å, HS	-1610.2202669135	-1610.60539465663	-1610.99171872331	-1611.37907052025
2.9 Å, LS	-1610.2215300658	-1610.59728131866	-1610.9746375486	-1611.35354408794
3.0 Å, HS	-1610.22235497658	-1610.60760914915	-1610.9937569479	-1611.38151452511
3.0 Å, LS	-1610.22070085199	-1610.59688653808	-1610.9746765613	-1611.35401470996
3.1 Å, HS	-1610.22167243693	-1610.60692282577	-1610.99334317653	-1611.38087573236
3.1 Å, LS	-1610.217934554	-1610.5945209746	-1610.97271333933	-1611.35245399318

3.4 Using CAM-B3LYP with (α_0, μ_0), (α_1, μ_0), (α_2, μ_0), (α_0, μ_1), (α_0, μ_2)

	CAM-B3LYP	(α_0, μ_1)	(α_0, μ_2)	(α_1, μ_0)	(α_2, μ_0)
2.5 Å, HS	-1611.89097577089	-1611.38747833672	-1611.34299951079	-1611.32222823265	-1611.39591141635
2.5 Å, LS	-1611.88502938301	-1611.38712082845	-1611.3352590941	-1611.32594234899	-1611.38469827048
2.6 Å, HS	-1611.90462900745	-1611.40066280693	-1611.35661406234	-1611.3354673646	-1611.40946764075
2.6 Å, LS	-1611.89623137055	-1611.39789258956	-1611.3464137077	-1611.33666790873	-1611.39587739764
2.7 Å, HS	-1611.9143714551	-1611.40998418537	-1611.36627554501	-1611.34482354735	-1611.41908369007
2.7 Å, LS	-1611.9033706846	-1611.40465986678	-1611.35349645168	-1611.34340933566	-1611.40296421048
2.8 Å, HS	-1611.92072680228	-1611.4160269421	-1611.3725759077	-1611.35090858822	-1611.4253387383
2.8 Å, LS	-1611.90750911106	-1611.40849385138	-1611.35757995123	-1611.34716155503	-1611.40710894382
2.9 Å, HS	-1611.92487100184	-1611.41991707505	-1611.37666955806	-1611.35481991459	-1611.42940894953
2.9 Å, LS	-1611.90921614652	-1611.40998443155	-1611.35926313759	-1611.34857109198	-1611.40886083442
3.0 Å, HS	-1611.92716104295	-1611.42202476781	-1611.37888774683	-1611.35693627744	-1611.43160961759
3.0 Å, LS	-1611.91037039356	-1611.41074158913	-1611.36034750735	-1611.34923305786	-1611.41001186591
3.1 Å, HS	-1611.92671882773	-1611.42158427981	-1611.37845776168	-1611.35648840979	-1611.43118697517
3.1 Å, LS	-1611.90942223563	-1611.40943538083	-1611.35931037198	-1611.34781841344	-1611.40905120747

3.5 Using PBE0r with 8, 9, 11 and 12.5% exact exchange

	PBE0r(8.00%)	PBE0r(9.00%)	PBE0r(11.00%)	PBE0r(12.50%)
2.5 Å, HS	-92.3344956	-92.3300055	-92.3233405	-92.320538
2.5 Å, LS	-92.3311828	-92.3232491	-92.3100367	-92.3026846
2.6 Å, HS	-92.3462091	-92.3414424	-92.3341483	-92.3308191
2.6 Å, LS	-92.3405257	-92.3323971	-92.3187345	-92.3109804
2.7 Å, HS	-92.3543663	-92.3493436	-92.3414466	-92.3376116
2.7 Å, LS	-92.3460165	-92.3377	-92.3235982	-92.3154653
2.8 Å, HS	-92.3588657	-92.3536212	-92.345276	-92.3410611
2.8 Å, LS	-92.3480944	-92.3396194	-92.3251619	-92.3167136
2.9 Å, HS	-92.3611994	-92.355807	-92.347126	-92.3426369
2.9 Å, LS	-92.3481381	-92.3395613	-92.3248698	-92.316218
3.0 Å, HS	-92.3628447	-92.3573201	-92.3483529	-92.3436116
3.0 Å, LS	-92.3474557	-92.3387272	-92.3236782	-92.3147042
3.1 Å, HS	-92.3612848	-92.3566503	-92.3474327	-92.3424879
3.1 Å, LS	-92.3447425	-92.3358379	-92.3203811	-92.3110351

4 Reference energies from the CCSD(T*)-F12B calculations on the $[Fe^{II}(NH_3)_6]^{2+}$ model complexes in kcal/mol

d_{N-N} / Å	$\Delta E_{\text{HL}}^{\text{UCCSD-F12B}}$	$\Delta E_{\text{HL}}^{\text{UCCSD(T*)-F12B}}$	$\Delta E_{\text{HL}}\,(\text{DK})$	$\Delta E_{\text{HL}}^{\text{UCCSD(T*)-F12B}} + \Delta E_{\text{HL}}\,(\text{DK})$
2.5	-16.9678	-7.0318	2.7643	-4.2675
2.6	-18.5519	-8.7567	2.7098	-6.0468
2.7	-20.1476	-10.4659	2.6602	-7.8057
2.8	-21.5542	-12.0082	2.5865	-9.4216
2.9	-23.0885	-13.6150	2.5470	-11.0680
3.0	-23.5710	-14.4150	2.3962	-12.0188
3.1	-23.8446	-15.1072	2.1977	-12.9095
